# The Effect of Cold Plasma Treatment on the Storage Stability of Mushrooms (*Agaricus bisporus*)

**DOI:** 10.3390/foods13213393

**Published:** 2024-10-25

**Authors:** Yalong Guo, Shuqiong Xia, Chong Shi, Ning Ma, Fei Pei, Wenjian Yang, Qiuhui Hu, Benard Muinde Kimatu, Donglu Fang

**Affiliations:** 1 College of Light Industry and Food Engineering, Nanjing Forestry University, Nanjing 210037, China; guoyalong@njfu.edu.cn (Y.G.); x16211357@163.com (S.X.); 2 State Key Laboratory of Tree Genetics and Breeding, Co-Innovation Center for Sustainable Forestry in Southern China, College of Forestry and Grassland, Nanjing Forestry University, Nanjing 210037, China; shichong0639@163.com; 3 Jiangsu Province Engineering Research Center of Edible Fungus Preservation and Intensive Processing, College of Food Science and Engineering, Nanjing University of Finance and Economics, Nanjing 210023, China; maning@nufe.edu.cn (N.M.); feipei87@163.com (F.P.); lingwentt@163.com (W.Y.); qiuhuihu@nufe.edu.cn (Q.H.); 4 Department of Dairy and Food Science and Technology, Egerton University, Egerton 20115, Kenya; muinde.bk@gmail.com

**Keywords:** *Agaricus bisporus*, cold plasma, response surface, optimization, postharvest quality

## Abstract

Postharvest *Agaricus bisporus* is susceptible to browning, water loss, and microbial infection. In order to extend its shelf life, cold plasma technology was used to treat and evaluate *A. bisporus*. Firstly, according to the results of a single factor test and response surface analysis, the optimal conditions for cold plasma treatment were determined as a voltage of 95 kV, a frequency of 130 Hz, and a processing time of 10 min. Secondly, storage experiments were carried out using the optimized cold plasma treatment. The results showed that the cold plasma treatment in the packaging significantly reduced the total viable count in *A. bisporus* by approximately 16.5%, maintained a browning degree at 26.9% lower than that of the control group, and a hardness at 25.6% higher than that of the control group. In addition, the cold plasma treatment also helped to preserve the vitamin C and total protein content of *A. bisporus*. In conclusion, cold plasma treatment showed great potential in enhancing the postharvest quality of fresh *A. bisporus.*

## 1. Introduction

*Agaricus bisporus*, commonly known as the white mushroom [1], is a fungus that has gained global attention due to its unique sensory and nutritional values [2]. However, *A. bisporus* has a short shelf life under natural postharvest conditions owing to its high water content, lack of protective surface cuticle, excessive respiration and transpiration, and microbial infection [3]. These factors contribute to the browning, water loss, softening, nutrient loss, and spoilage of postharvest *A. bisporus*. In recent years, various preservation methods have been explored to address these challenges, including improved packaging techniques [4,5,6], light treatment [7], and irradiation treatment [8]. However, these methods have limitations such as the complexity in preparing active packaging, the high cost of raw materials, and difficulties in mass production. Alternatively, single low-temperature preservation methods require significant investment and have low efficiency. Therefore, researchers have increasingly focused on developing new preservation technologies, such as cold plasma combined with a refrigerated packing treatment, which have shown promising results in the preservation and processing of various foods [9,10].

Cold plasma is a novel non-contact cold sterilization technology that exhibits broad-spectrum sterilization properties [11]. Its bactericidal efficacy has been demonstrated in various food products including *Flammulina velutipes* [12], *Agaricus bisporus* [13], winter jujubes [14], apricot [15], and fresh wolfberries [16]. Plasma, a highly active substance rich in reactive oxygen and nitrogen [17], can permeate the microbial cell membrane, leading to DNA damage and a reduction in the activity of cell-degrading enzymes [18]. Moreover, these active substances also impact on the quality and properties of biomolecules [17]. For instance, cold plasma has the ability to depolymerize amylose [19], disrupt peptide bonds in proteins [20], and promote lipid oxidation [21].

Cold plasma can be generated using various methods. Among these methods, dielectric barrier discharge (DBD) is particularly well-suited for food industrial applications [22]. DBD offers several advantages, including the ability to produce large-area and uniform plasma, short processing times, high energy efficiency, high antimicrobial efficiency, and minimal impact on food quality and the environment [23]. However, due to insufficient research on cold plasma technology and the lack of theoretical foundations, its application in the field of food preservation is not widespread, especially for mushroom preservation.

This study aimed to improve the storage period of *A. bisporus* by optimizing the treatment conditions of cold plasma and investigating its effects on the postharvest physiological and nutritional quality of the mushroom. The effects of cold plasma voltage, frequency, and processing time on the surface microorganisms and color were examined during the storage of *A. bisporus* using the Box-Behnken response surface method and a quadratic polynomial regression model. Additionally, the postharvest physiological characteristics and nutritional changes in *A. bisporus* after cold plasma treatment were evaluated. These findings would provide valuable insights into the application of cold plasma treatment on edible mushrooms.

## 2. Materials and Methods

### 2.1. Sample Source and Pre-Treatment

*A. bisporus* samples were provided by Jiangsu zhongyouxinghe mushroom technology Co., Ltd. (Lianyungang, China) and promptly sent to the laboratory within 2 h of harvest. To ensure the freshness of the edible fungi, only unopened mushrooms without any mechanical damage were chosen. The selected mushrooms had a cap diameter of approximately 40 mm and weighed between 18–25 g. Prior to testing, the *A. bisporus* samples were pre-cooled at 4 °C and 90% relative humidity for 24 h. The selected *A. bisporus* samples were randomly packed, with each box containing approximately 120 g. The boxes were placed in food-grade polypropylene PP boxes measuring 18 cm × 13 cm × 6 cm.

### 2.2. Equipment and Reagents

This study used cold plasma equipment from Nanjing Suman (CPS-1, Nanjing Suman Plasma Technology Co., Ltd., Nanjing, China). *A. bisporus* was placed on a glass plate (dielectric barrier layer) among the electrodes. The size of electrodes was 17 cm × 13 cm.

Folinol and gallic acid (CAS: 149-91-7) were purchased from Shanghai yuanye Bio-Technology Co., Ltd. (Shanghai, China). Guaiacol (CAS: 90-05-1), phthalic acid, diphenols, ergosterol (CAS: 57-87-4), and vitamin D2 (CAS: 50-14-6) were purchased from Shanghai Macklin Biochemical Technology Co., Ltd. (Shanghai, China). Methanol and other reagents were purchased from Sinopharm chemical reagent Co., Ltd. (Shanghai, China). Except for the liquid chromatography-specific reagents, these reagents were of analytical grade and used without further purification.

### 2.3. Cold Plasma Process Optimization

#### 2.3.1. Single Factor Experiment

As shown in Figure S1A,B, *A. bisporus* was sealed in a polyethylene film bag with a thickness of 0.07 mm. It was then promptly placed on the electrode of a cold plasma device for further processing. During the experiment, some fixed parameters were set as follows: the number of discharges was once, the distance between the plates was 60 mm, the upper limit of the current was 1.5 A, and the temperature was room temperature: 25 ± 5 °C. When the single factor experiment was carried out, the other factors were fixed as follows: treatment voltage 125 kV, treatment frequency 145 Hz, processing time 7 min.

This study was based on the cold plasma treatment voltage (50, 65, 80, 95, 110, 125, 140, 155 kV), treatment frequency (70, 95, 120, 145, 170, 195 Hz) and processing time (1, 4, 7, 10, 13, 16 min) as the optimized conditions, and the samples were processed in the sealed box. Each treatment was repeated three times. *A. bisporus* without cold plasma treatment was used as a control. After treatment, *A. bisporus* was placed in a constant temperature and humidity box at 4 °C and 90% relative humidity and stored for 24 h. Please refer to the methods described in Section 2.5.2 and Section 2.5.3 for the determination of sterilization rate and color difference. 

For the detailed results in the single factor experiment, please refer to the Supplementary Information and Figures S2–S4.

#### 2.3.2. Box-Behnken Experiment for Optimization

The optimization of the cold plasma treatment conditions for *A. bisporus* was conducted using the Box-Behnken design. The variables considered were voltage, frequency, and time, while the responses measured were the surface L* of *A. bisporus* and the sterilization rate, denoted as Y. Prior to the experiment, the range of each variable was determined through a single factor experiment, and other parameters of the equipment were the same as shown in Section 2.3.1. A total of seventeen experiments were performed based on the Box-Behnken design matrix, as detailed in Table S1. In each experimental group, *A. bisporus* samples were treated with cold plasma using the specified voltage, frequency, and time settings. Subsequently, the surface L* and sterilization rate were measured after the treatment.

### 2.4. Cold Plasma Treatment and Storage of A. bisporus

As shown in Section 2.1, the packaged *A. bisporus* were randomly divided into four groups (Table 1 and Figure S1). They were the following four groups: cold plasma treatment combined with PE sealed packaging (CP+PE), PE sealed packaging (PE), direct cold plasma treatment (CP), and control group (Control). The packaging group used 0.07 mm thick polyethylene film bags for the sealed packaging. Samples were stored at 4 °C and 90% relative humidity for 12 days, and the physical and chemical indicators were measured and evaluated on days 0, 3, 6, 9 and 12 [24].

### 2.5. Preservation Quality Investigation

#### 2.5.1. Hardness, Weight Loss Rate, and Moisture Content

The hardness was measured using a fruit firmness tester with a 3.5 mm flat probe (GY-4; Jinkelida Instrument Co., Ltd., Beijing, China) and expressed as N [25].

Weight loss was measured by weighing samples before and after storage. The results were expressed as the percentage of weight lost relative to the initial weight.

The moisture content was determined by a direct drying method. First, the weighing bottle was dried to a constant weight and the weight (W_1_) was recorded. Secondly, approximately 5 g of chopped mushroom samples were added, and the total weight (W_2_) of the sample and the weighing bottle was recorded in detail. Finally, the sample was dried to a constant weight and the total weight (W_3_) after drying was recorded. The formula for moisture content is as follows:

$$X\left(\%\right)=\frac{W_{2}-W_{3}}{W_{2}-W_{1}}\times 100\%$$

#### 2.5.2. Microbial Colony Assay of Mushroom Epidermis

The total number of colonies was measured according to a previously described method with minor modifications [8]. *A. bisporus* (25 g) from each group were placed into a sterile conical flask, to which sterile saline (225 mL) was added. Then, the solution was vortexed for 2 min. The solution (100 μL) was then deposited onto plate count agar plates and evenly spread and incubated for 48 h at 37 °C for the aerobic colony count. Each sample had three replicates. The results were expressed as the number of colonies per gram of fresh weight (lg CFU g^−1^), and lg was logarithmic to the base of 10.

$$Y(\%)=(1-\frac{M_{1}}{M_{2}})\times 100\%$$

Y(%) was the sterilization rate.

M_1_ was the number of colonies after low temperature plasma treatment.

M_2_ was the number of untreated colonies.

#### 2.5.3. Mushroom Appearance, Color, Peroxidase, and Polyphenol Oxidase

The mushrooms were photographed at the beginning and at the end of the storage period [26]. The color was recorded as L*, a*, and b* using a 3NH colorimeter (3NH SR-66; Shenzhen 3NH Technology Co., Ltd., Shenzhen, China). The browning index (BI) was determined according to the following formula:

(1) BI=[100×(X−0.31)]

(2) $$X=\frac{a*-1.75L*}{5.546L*+a*-3.012b*}$$

To determine enzyme activities, the mushrooms (5 g) were added to 5 mL of extraction buffer containing 1 mmol L^−1^ IPEG, 4% PVPP, and 1% Triton X-100. The extract was centrifuged at 4 °C, 12,000× *g* for 30 min.

Peroxidase (POD) activity was measured according to the method of Yan with some modifications [5]. Firstly, 0.5 mL of the enzyme extract was mixed with 3.0 mL of 25 mmol L^−1^ guaiacol, and afterwards 200 µL of 0.5 mol L^−1^ H_2_O_2_ was added and quickly mixed. The absorbance of the reaction mixture was then measured six times at 470 nm with 1 min intervals. One unit (U) of POD activity was defined as a change of 0.01 at 470 nm per minute. POD activity was expressed as U g^−1^ fresh weight.

Polyphenol oxidase (PPO) was measured according to the method of Yan [5] with some modifications. Firstly, 4.0 mL of 50 mmol L^−1^ sodium acetate buffer solution and 1.0 mL of 50 mmol L^−1^ catechol were mixed, and then 100 µL of the enzyme extract was added and mixed well. The absorbance of the reaction mixture was finally measured six times at 420 nm with 1 min intervals. One unit (U) of PPO activity was defined as a change of 0.01 at 420 nm per minute. PPO activity was expressed as U g^−1^ fresh weight.

#### 2.5.4. Vitamin C, Total Protein Content, Total Phenolic Content, and Total Soluble Solids Content

The amount of vitamin C and total protein were determined using kits from Nanjing Jiancheng Biotechnology Co., Ltd. (Nanjing, China).

The total phenolic content (TPC) of the samples was detected by the Folin-Ciocalteu method [24], and gallic acid was used to construct a standard curve. Absorbance of the supernatant was measured at 760 nm and the mass fraction of total phenol in the samples (mg g^−1^) was calculated.

The determination of total soluble solids (TSS) was based on Ni’s method [27] with some minor modifications. The mushroom samples (10 g) were added to 15 mL of deionized water (pH 7), then ground in a mortar and filtered with four layers of gauze to remove the filter residue. Subsequently, TSS (%) was measured via a refractometer (Atago PAL-1; Atago Co. Ltd., Tokyo, Japan) using the supernatant.

#### 2.5.5. Determination of Vitamin D and Ergosterol

A direct extraction method was employed to extract vitamin D and ergosterol from the mushrooms (Nzekoue et al., 2022) [28]. Mushroom powder (0.2 g) was subjected to ultrasonic extraction in ethanol (5 mL) for 30 min. The ultrasonic extraction conditions included a power of 240 W, frequency of 40 kHz, and temperature of 40 °C. Following ultrasonication, the extract was filtered using a 0.45 μm organic microporous membrane and transferred to a liquid phase test bottle for subsequent HPLC analysis.

The HPLC system (Waters alliance e2695 Series, Milford, MA, USA) was equipped with a quaternary pump, on-line degasser, thermostatic autosampler, and diode array detector (DAD). The chromatographic separation was performed on a Gemini C18 analytical column (250 × 3.0 mm, 5 μm) preceded by a security guard column C18 (4 × 3 mm, 5 μm). The mobile phase was A phase composed of water and B phase composed of methanol, and the flow rate was 0.8 mL min^−1^. The gradient elution was started with 80% B phase for 5 min, increased to 100% in 6 min, maintained for 13 min, and returned to the initial conditions within 1 min. The injection volume was 20 μL and the column temperature was 40 °C. Vitamin D_2_ and ergosterol were detected at 265 nm and 280 nm, respectively, and used as a calibration curve for quantitation.

### 2.6. Statistical Analyses

All indicators were measured in triplicate. The data were expressed as mean ± SD, and the experimental data were organized and plotted using Origin Pro 2024b (OriginLab, Northampton, MA, USA). Analysis of variance (ANOVA) was performed using SPSS 26 software (IBM, Armonk, NY, USA). To determine the statistical differences, comparisons of the means between the controls and the treatments were performed using Duncans test at a significance level of *p* < 0.05.

## 3. Results and Discussion

### 3.1. Optimization of Cold Plasma Treatment for A. bisporus

High-intensity treatments have been found to potentially disrupt the surface structure of agricultural products and oxidize antioxidant substances [16]. In this study, we aimed to optimize the treatment conditions using a response surface methodology. It is important to consider that voltage can cause air discharge effects, frequency may impact energy input and product structure, and processing time determines the total treatment energy during cold plasma processing [29]. Therefore, we selected voltage, frequency, and time as the main factors for the response surface analysis. Our goal was to find suitable treatment conditions that effectively kill pathogenic microorganisms while minimizing browning and nutritional damage in *A. bisporus.* To prepare for the response surface analysis, we conducted a preliminary single factor experiment to assess the influence of voltage, frequency, and time on the surface L* and sterilization rate (Y) of *A. bisporus* (Figures S1–S3). Table S1 presented the design and results of the Box-Behnken experiments. Using DesignExpert 8.0 software, we performed quadratic polynomial regression fitting of the sterilization rate data in Table S1 to obtain a multiple regression equation describing the relationship between the sterilization rate and the independent variables.

In Table 2, the L* regression model is not significant, and the sterilization rate Y regression model is extremely significant (*p* < 0.01). The lack of fit term of the sterilization rate Y regression model is not significant (*p* = 0.6966 > 0.05), and the coefficient of determination (R^2^ = 0.9631) and the corrected coefficient of determination (R^2^_Adj_ = 0.9156) of the equation is high, indicating that the fitting degree of the model is high and the experimental error is small.

**Figure 1:**
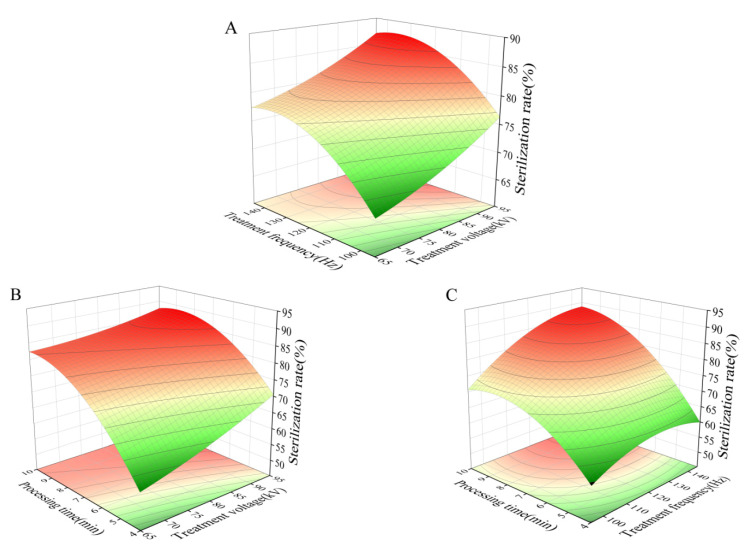
Response surface diagrams showing the interactions of different factors on the sterilization rate. (**A**) Effects of treatment voltage and frequency on the sterilization rate of *A. bisporus*. (**B**) Effects of treatment voltage and time on the sterilization rate of *A. bisporus*. (**C**) Effects of treatment time and frequency on the sterilization rate of *A. bisporus*.

Improper handling conditions can have a significant impact on the freshness of mushrooms by promoting biological processes like respiration, ripening, and senescence, and enabling pathogenic microorganisms. To analyze the impact of different experimental factors on the response variables, we considered the processing time, treatment frequency, and voltage. The F values indicated the importance of each factor, with processing time having the least influence, followed by treatment frequency, and voltage being the most influential. In Figure 1A–C, we present three-dimensional response surface diagrams to visualize the interaction of these factors with the response variables. The shape of the response surface indicates the extent of interaction, with a greater curvature suggesting a more significant interaction.

The predicted optimal treatment conditions were determined based on the highest bactericidal rate Y. The predicted optimum treatment conditions were 95.154 kV, 130.41 Hz, and 9.612 min, adjusted to 95 kV, 130 Hz, and 10 min for operational purposes. These conditions were verified, and the actual Y was 89.05% with an average relative error of ±1.744%, indicating reliable model optimization results. Many studies have reported the effects of treatment parameters on the preservation quality of edible fungi. Excessive application of parameters usually caused higher oxidative damage to mushrooms and reduced the edible quality of mushrooms [19].

### 3.2. Postharvest Preservation of A. bisporus

#### 3.2.1. Hardness, Weight Loss Rate, Moisture Content, and Total Number of Bacterial Colonies

**Figure 2:**
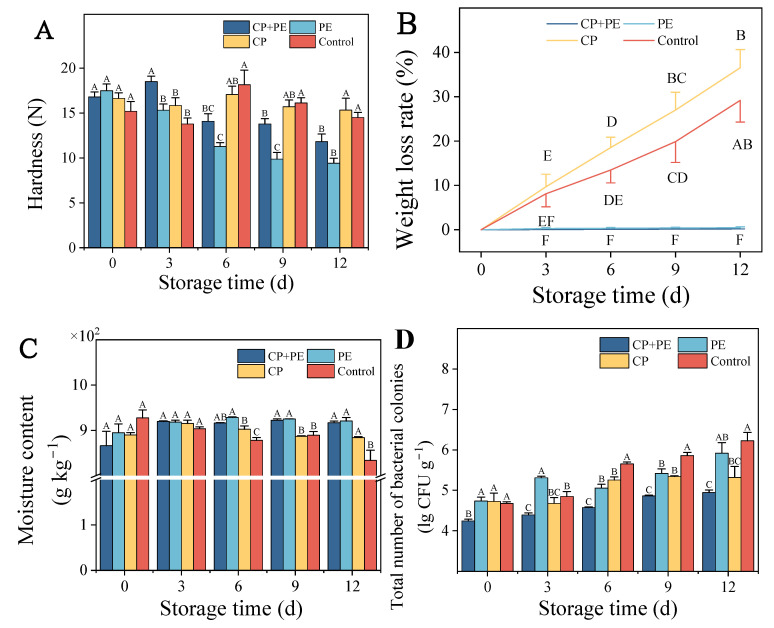
Effects of different treatments on hardness (**A**), weight loss rate (**B**), moisture content (**C**), and total number of colonies (**D**) of *Agaricus bisporus.* Distinct letters demonstrate a significant difference between different treatments (*p* < 0.05).

According to Figure 2A, the polyethylene packaging group gradually softens throughout the postharvest storage process, while the unpackaged group remain unchanged in hardness. This could be due to the severe water loss and shrinkage of the mushroom surface in the unpackaged group, resulting in the formation of a dense waterproof layer on the mushroom surface. On the ninth day of storage, the hardness of the CP+PE group remained at 13.79 N, which was significantly higher (*p* < 0.05) than that of the PE group. This indicated that the cold plasma treatment had the potential to delay the softening of the mushrooms [30]. This delay in softening might be due to the inactivation of bacteria by active plasma species, inhibition of enzyme activity, and a reduction in the respiratory rate [13].

The rate of water transpiration and the respiration rate in mushrooms during storage are two common issues leading to weight loss [31]. In Figure 2B, the weight loss in unpackaged mushrooms significantly increases (*p* < 0.05) with an extended storage time. After three days of storage, the weight loss in samples from the CP and control groups exceeded the permitted limit, with values of 9.68% and 8.11%, respectively. Mushrooms were considered to have lost their value when their water loss reached 5% [32]. However, we observed condensation on the surface and inside of the mushrooms in the packaging group, which might be attributed to the low water vapor permeability of the low-density polyethylene film. This condensation phenomenon could potentially contribute to the deterioration of the mushrooms [33]. The moisture content of *A. bisporus* is an important indicator of freshness. Figure 2C demonstrates that the moisture content of unpackaged mushrooms decreases after 12 days of storage, consistent with the results of the weight loss rate.

In this study, the total number of bacteria was used to monitor the bactericidal effect of cold plasma on *A. bisporus*. The results in Figure 2D demonstrate an upward trend in the number of microorganisms during the storage period. Towards the later stage of storage, the CP+PE group exhibited significantly lower microorganism counts compared to the PE group (*p* < 0.05). These findings indicate that the cold plasma treatment effectively reduces microorganism levels on the surface of *A. bisporus*, thereby enhancing mushroom safety. In addition, similar results were also exhibited in the research of Subrahmanyam, K, which showed that the number of bacteria and fungi in *A. bisporus* decreased significantly after cold plasma treatment (*p* < 0.05) [13]. Pourbagher, R used surface dielectric barrier discharge plasma to inactivate *P. tolaasii Pt18* bacteria inoculated into *A. bisporus*. H_2_O_2_ + air gas had the greatest effect on reducing bacteria, and the number of colonies decreased to 4.23 lg CFU g^−1^ on the 21st day of storage [34]. It was worth noting that the bactericidal performance of cold plasma was attributed to various active substances which caused lipid peroxidation, enzyme inactivation, and DNA degradation, eventually leading to microbial inactivation [35].

#### 3.2.2. Appearance, Color, Peroxidase, and Polyphenol Oxidase

**Figure 3:**
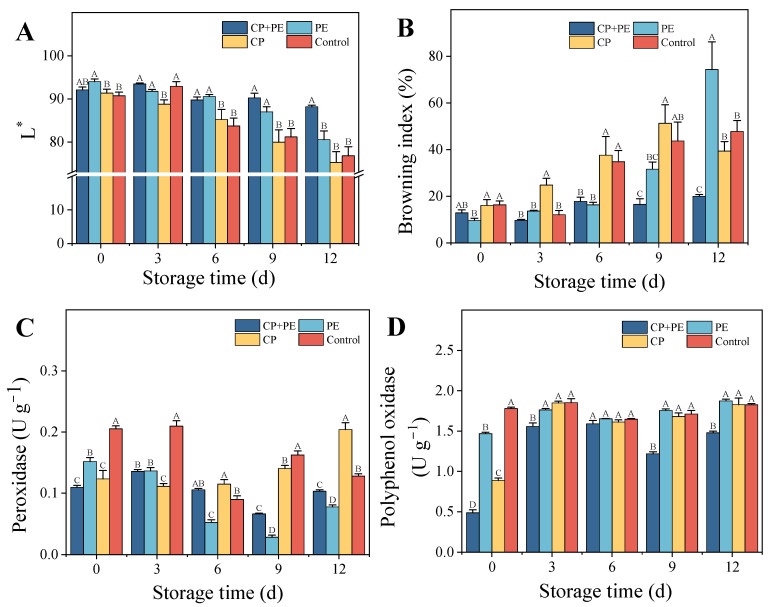
Effects of different treatments on L* (**A**), browning index (**B**), polyphenol oxidase (**C**), and peroxidase (**D**) of *Agaricus bisporus.* Distinct letters demonstrate a significant difference between different treatments (*p* < 0.05).

**Figure 4:**
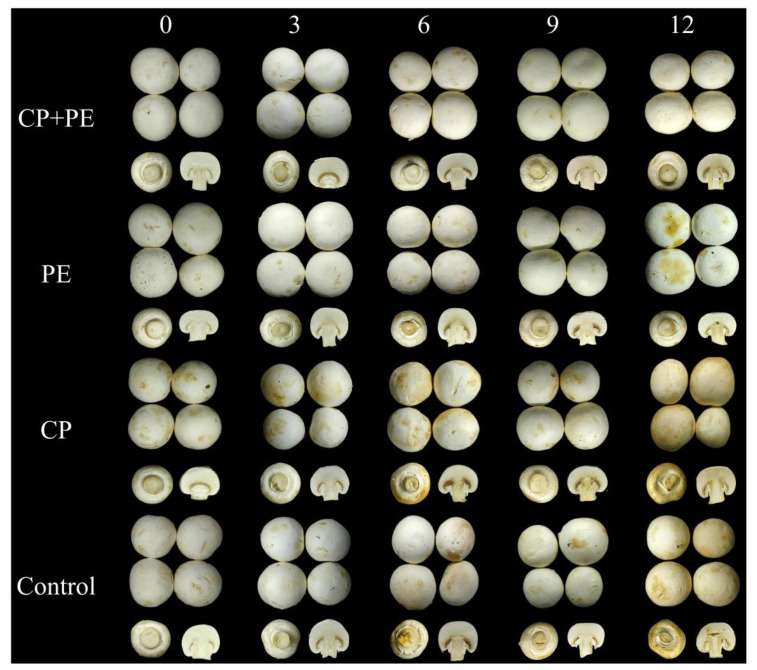
Effects of different treatments on the appearance of *Agaricus bisporus*.

Changes in the mushroom surface color play a crucial role in determining the shelf life of *A. bisporus*, and are a significant factor in influencing consumer acceptance [27,36]. A decrease in the L* value indicates a darker colored *A. bisporus.* Figure 3A highlights an important pattern where the L* value of *A. bisporus* progressively decreases with a longer storage time. The slowest color change is observed in *A. bisporus* treated with cold plasma and packed in polyethylene. These color change indices are further supported by the mushroom appearance images at different storage periods (Figure 4). After nine days of cold storage, the PE group samples exhibit black spots and noticeable browning, whereas the CP+PE treated group maintain a better color (Figure 3B). Cold plasma treatment in packaging effectively inhibited browning. Zhao’s research indicates that plasma activated water (PAW) treatment could effectively suppress browning and retain the desired visual appearance of button mushrooms [37]. The increase in browning values and the decrease in lightness values of mushrooms could be due to microbial growth, water loss, and enzymatic browning [13,35].

Enzymatic browning is the main type of *A. bisporus* browning, which is generally considered to be brown spots formed due to the oxidation of phenols by PPO and POD [38]. Cold plasma treatment has been shown to inactivate enzymes [39]. Following the initial treatment on day 0, the activities of peroxidase (POD) and polyphenol oxidase (PPO) in the cold plasma treatment group are significantly decreased (Figure 3C,D). This decrease in enzyme activity could be attributed to a reduction in the α-helix structure and an increase in the β-sheet structure within the enzyme proteins’ secondary structure [40]. However, during the later storage period, this trend is reversed. The POD activity in the CP+PE group is significantly higher than that of PE group (*p* < 0.05), whereas the PPO activity is significantly lower (*p* < 0.05). Roghayeh Pourbagher’s research indicated that, under dual dielectric barrier discharge plasma treatment, the POD activity in *A. bisporus* decreased, and this decreasing trend aligned with the inactivation trend of PPO [41]. Both enzymes decreased with an increase in processing time. This finding is consistent with the trend observed in *A. bisporus* that we processed on day 0. However, as the storage time increases, the POD activity in the CP+PE treatment group is significantly higher than that in the PE group. This discrepancy may be related to the equipment parameters used, specifically their design, with a fixed frequency of up to 6200 Hz. Previous studies indicated that low-power and short-term cold plasma treatment did not decrease POD activity, but rather enhanced it [42]. This finding was confirmed by the preservation of acai pulp and blueberries [43,44]. However, the exact mechanism underlying the activity of the POD enzyme remained unclear [45].

#### 3.2.3. Vitamin C, Protein Content, Total Phenolic Content, and Total Soluble Solids

**Figure 5:**
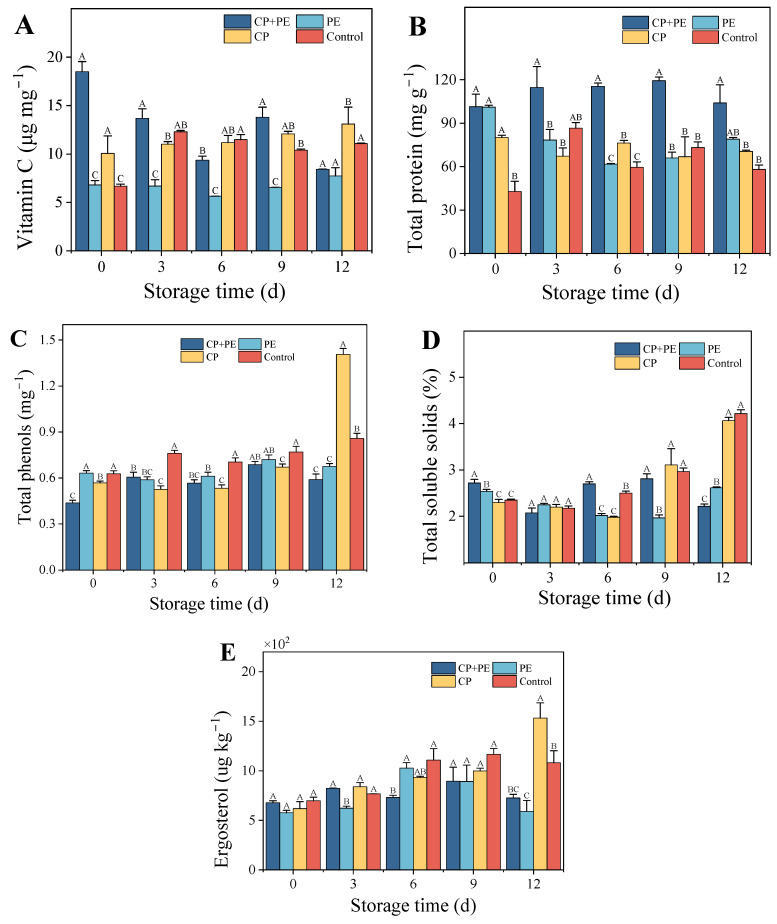
Effects of different treatments on vitamin C (**A**), total protein content (**B**), total phenols (**C**), total soluble solids (**D**), and ergosterol (**E**) of *Agaricus bisporus*. Distinct letters demonstrate a significant difference between different treatments (*p* < 0.05).

Vitamin C, an essential nutrient, plays a crucial role in protecting DNA from the damage caused by free radicals [46]. The vitamin C content of the CP+PE group decreases during storage (Figure 5A). This decline could be attributed to the production of numerous active substances by the cold plasma treatment, prompting *A. bisporus* to generate more vitamin C as a defense mechanism against oxidative damage. Over time, the active substances in the packaging gradually decomposed, leading to a stabilization of the vitamin C content in *A. bisporus*. On the other hand, the CP and control groups, being exposed to the external environment and environmental microorganisms, consistently maintained high levels of vitamin C. The vitamin C content in the PE group remained relatively stable throughout. The treatment of cashew apple juice with cold plasma resulted in an increase in the vitamin C content [47], which was considered beneficial. This increase could be attributed to the activation of the enzyme dehydroascorbate reductase, which converts dehydroascorbic acid back to ascorbic acid through the ascorbate–glutathione cycle. Similarly, in a study on bananas, cold plasma treatment caused an increase in the vitamin B_6_ content, as the treatment disrupted the cell wall and released the contents [48]. In Ding’s study, cold plasma treatment could effectively maintain the vitamin C content of *Flammulina velutipes* during storage, which was significantly higher than that of the control group (*p* < 0.05) [12]. However, Kadavakollu Subrahmanyam’s study pointed out that during the storage period, there were no significant differences in the vitamin C content between different treatment groups (*p* > 0.05) [13]. The difference in this result may be related to the strength of the treatment parameters.

Protein content plays a significant role in assessing the nutritional value of mushrooms. In fact, mushroom protein is considered to be of a higher nutritional quality compared to plant protein [49]. The proteins present in mushrooms are involved in various processes such as growth, maturation, senescence, disease resistance, and stress resistance. Figure 5B demonstrates that the soluble protein content in the CP+PE group initially increased and then stabilized, maintaining a higher content throughout the storage period. However, the CP and control groups experienced severe browning and water loss, leading to protein decomposition. The soluble protein content remained relatively low throughout the storage period. In the case of the PE group, the presence of microorganisms resulted in the consumption of protein as a nutrient. It is worth noting that the impact of cold plasma treatment on the structure and function of proteins is well-documented. However, there is a scarcity of literature comparing and explaining the effects of cold plasma on proteins in edible fungi substrates. In this study, cold plasma treatment was found to increase the protein content, which aligned with previous studies on soybean sprouts and black gram seeds [50,51]. This increase could be attributed to the induction of free radicals and ozone oxidation, which promote chemical reactions between amino acid side chains and protein backbones, ultimately resulting in the polymerization of small-molecule proteins [52].

Total phenols are considered beneficial antioxidants due to their ability to scavenge harmful active oxygen species [53]. Figure 5C shows the change in total phenolic content (TPC) during the entire storage period. The CP+PE group exhibits the lowest TPC on day 0. On the ninth day of storage, the PE group reaches its peak TPC. The CP group reaches its highest TPC on the 12th day. Cold plasma treatment could decrease the total phenol content. This decrease is attributed to the ability of phenolic compounds to scavenge free radicals and the reaction of reactive oxygen species with the plasma [43]. Higher treatment intensity leads to the production of ozone and singlet oxygen, which could cleave the central heterocyclic ring in the polyphenol skeleton, leading to a decrease in TPC.

TSS represents the sugar content in the harvested mushrooms, which typically decreases during storage [54]. Limiting the use of soluble solids can help prolong the shelf life of the product. The findings presented in Figure 5D demonstrate noticeable differences in the percentage of soluble solids between packaged and unpackaged mushrooms, which is consistent with the observed changes in the vitamin C content. We obtained similar results in the study of *Flammulina velutipes* [12]. In the packaging group, the TSS content initially decreased and then increased. Before the ninth day of storage, the CP+PE group had a higher TSS content than the PE group, possibly due to a higher microbial content in the PE group, leading to TTS decomposition. On the other hand, the TSS content in the unpackaged group showed a consistent increase throughout the storage period, which might serve as an indicator of mushroom maturity [33].

#### 3.2.4. Vitamin D and Ergosterol

Vitamin D deficiency is a global concern for most vegetarians, as most vitamin D-rich foods are derived from animals. Under ultraviolet irradiation, ergosterol is photolyzed to produce a vitamin D precursor, and then slowly isomerized to vitamin D by a thermal reaction. Ergosterol is an important part of the fungal cell membrane, and its chemical structure is similar to cholesterol. It has been observed that ergosterol can enhance the cell membrane of mushrooms, regulate membrane fluidity, and assist membrane transportation, which is similar to animal cholesterol [55]. The free and esterified forms of ergosterol play an important role in membrane fluidity and integrity. Therefore, ergosterol is a target for a variety of antifungal drugs [3].

According to the previous research results, which showed that the vitamin D content in the cultivated *A. bisporus* is very low [56]. Generally, when air or oxygen was used as the feed gas, ozone was a key substance for the efficiency of cold plasma. At this time, the ultraviolet radiation power generated by cold plasma was very small, making it impossible for ergosterol in mushrooms to be converted into vitamin D through ultraviolet radiation. Although, the reactive oxygen species (ROS) and reactive nitrogen species (RNS) produced by cold plasma could cause cell damage, but this was not the same as the effects of ultraviolet radiation [47,57,58]. we did not find the presence of vitamin D in *A. bisporus* that was treated with cold plasma, which might explain the research results.

In Figure 5E, it is observed that the ergosterol content of *A.bisporus* in the CP and control groups follows a gradually increasing trend as the storage period progresses. On the last day of storage, it is significantly higher than in the other two treatment groups (*p* < 0.05). Since the mushrooms used in this experiment were freshly harvested, the mushrooms still have biological activity and metabolic capacity. Mushrooms are known for their high metabolic capacity and growth rate; induction of ergosterol synthesis following prolonged exposed storage may have occurred [59].

On the third day of storage, the ergosterol content in *A. bisporus* treated with cold plasma was higher than that in the PE group (*p* < 0.05). However, on the sixth day, the ergosterol content in the PE group increased by about 65%. This was different from the study of traditional cold plasma treatment of fungi and also different from the results obtained in the treatment of non-edible fungi with cold plasma. *A. bisporus* is a large fungi and tends to have more complex cell wall structures, which leads to this difference [60,61]. The content of ergosterol in CP+PE group did not increase, which may be due to the oxidative stress on the mushroom cell membrane caused by cold plasma treatment, resulting in the ergosterol participating in antioxidant metabolism. At the later stage of storage, ROS and RNS in the packaging group decreased, and there was no significant difference in ergosterol content in the packaging group (*p* > 0.05).

#### 3.2.5. Correlation Analysis and PLS-DA

**Figure 6:**
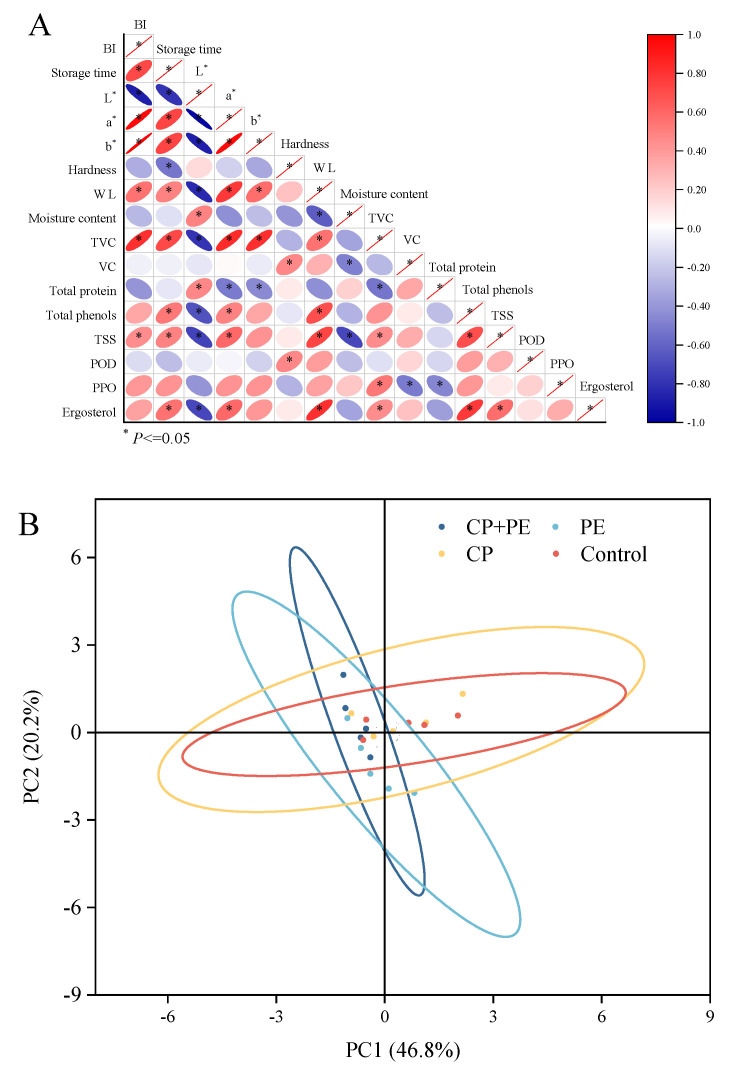
The correlation analysis (**A**) and principal component analysis (**B**).

The biochemical characteristics and sensory characteristics of *A. bisporus* undergo different degrees of change during storage. Figure 6A shows the correlation analysis of the indicators of *A. bisporus* between different treatment groups. Storage time is significantly correlated with browning degree, hardness, weight loss rate, total number of colonies, and soluble solids. The changes of L* and a* are also significantly correlated with total phenol and total protein. However, no significant correlation was found with the changes in PPO and POD enzymes, which indicates that cold plasma treatment regulates the changes in the factors leading to browning. Figure 6B shows the principal component analysis of the effects of different treatment methods on the quality of *A. bisporus* during storage. The pre-member rates of the first and second principal components were 46.8% and 22.2%, respectively. During the storage of *A. bisporus*, the soluble solids content, weight loss rate, browning degree, and total number of colonies contributed more to the first and second principal components. Furthermore, ergosterol, total phenol, total protein, and the water content change index contributed more to the first principal component, while hardness and vitamin C contributed more to the second principal component. Therefore, soluble solids content, weight loss rate, browning degree, and the total number of colonies were the key indicators during the storage of *A. bisporus.* With an increase in storage time, the CP and control groups moved to the first quadrant due to the lack of packaging protection, which was represented by the serious water loss and browning of the mushrooms. The PE group moved to the lower half of the storage period until it appeared in the fourth quadrant, indicating that the *A. bisporus* was infected with microorganisms and accompanied by browning at this time. The CP+PE group represented better quality, which manifested as two aspects. Firstly, there was no serious browning and water loss. Due to the bactericidal effect of the reactive oxygen species, there was no large-scale microbial infection in the packaging. On the other hand, the nutrients in *A. bisporus* did not suffer serious losses and remained relatively stable. This was similar to the results of the correlation analysis, which proved that the cold plasma treatment in the packaging affected the storage process of *A. bisporus* by increasing the sterility and reducing water loss.

## 4. Conclusions

This study determined the optimal conditions for cold plasma treatment (95 kV, 130 Hz, 10 min) and evaluated its impact on *A. bisporus* quality during storage. The CP+PE group effectively reduced the presence of spoilage microorganisms and minimized the risk of contamination post-treatment, while simultaneously preserving key quality characteristics such as hardness, weight loss rate, moisture content, and browning. Furthermore, the treatment minimized the loss of total protein and TSS in postharvest *A. bisporus*. In contrast, the PE packaging group exhibited severe browning, higher microbial growth, and lower vitamin C content during storage. Interestingly, the treatment reduced the activity of the PPO enzyme but stimulated the activity of POD, which contributed to maintaining the color of *A. bisporus*. These results were further supported by correlation and principal component analyses. Overall, this study highlighted the promising application of optimized cold plasma technology in eliminating pathogenic microorganisms and ensuring stable quality in fresh agricultural products. It also provided a valuable research foundation for the use of cold plasma technology in the storage and preservation of such products.

## Figures and Tables

**Table 1:** Process conditions for the treatment of *Agaricus bisporus*.

Treatment	Packaging	Plasma Treatment
CP+PE	Yes	Yes
PE	Yes	No
CP	No	Yes
Control	No	No

**Table 2:** Analysis of variance of regression equations for two response variables.

Source	Y			L*		
	F-Value	*p*-Value		F-Value	*p*-Value	
Model	20.29	0.0003	significant	1.39	0.3079	not significant
A-A	20.76	0.0026		5.19	0.0459	
B-B	25.24	0.0015		0.0004	0.9851	
C-C	99.1	<0.0001		0.0003	0.9857	
AB	0.0028	0.9589		1.26	0.2887	
AC	2.19	0.1826		0.8428	0.3802	
BC	5.03	0.0597		1.04	0.3316	
A^2^	0.5301	0.4902				
B^2^	7.97	0.0257				
C^2^	20.74	0.0026				
Lack of Fit	0.51	0.6966	not significant	0.0411	0.9992	not significant
R^2^	0.9631			0.4544		
Adjusted R^2^	0.9156			0.1271		
Predicted R^2^	0.7948			0.2984		

## Data Availability

The original contributions presented in the study are included in the article/Supplementary Materials, further inquiries can be directed to the corresponding author.

## Supplementary Materials

The following supporting information can be downloaded at: https://www.mdpi.com/article/10.3390/foods13213393/s1, Figure S1. Schematic diagram of Agaricus bisporus storage box. Figure S2. Effects of different treatment voltages on the whiteness (A), total number of colonies and sterilization rate (B), appearance (C) of *Agaricus bisporus*. Figure S3. Effects of different treatment frequencies on the whiteness (A), total number of colonies and sterilization rate (B), appearance (C) of *Agaricus bisporus*. Figure S4. Effects of different processing time on the whiteness (A), total number of colonies and sterilization rate (B), appearance (C) of *Agaricus bisporus*. Table S1. Experiment design and results for response surface analysis.
