# Supplementary material for: The Effect of Cold Plasma Treatment on the Storage Stability of Mushrooms (Agaricus bisporus)
Source: Foods. 2024 Oct 25;13(21):3393. doi: 10.3390/foods13213393 (PMC11545018; doi:10.3390/foods13213393)
Supplement: Supplementary file 1 [file foods-13-03393-s001.zip › foods-3238822-supplementary.pdf]

Supporting information

The Effect of Cold Plasma Treatment on the Storage Stability of Mushrooms (*Agaricus bisporus*)

Yalong Guo [1], Shuqiong Xia [1], Chong Shi [2], Ning Ma [3], Fei Pei [3], Wenjian Yang [3], Qiuhui Hu [3], Benard Muinde Kimatu [4] and Donglu Fang [2,*]

[1] College of Light Industry and Food Engineering, Nanjing Forestry University, Nanjing 210037, China
[2] State Key Laboratory of Tree Genetics and Breeding, Co-Innovation Center for Sustainable Forestry in Southern China, College of Forestry and Grassland, Nanjing Forestry University, Nanjing 210037, China
[3] Jiangsu Province Engineering Research Center of Edible Fungus Preservation and Intensive Processing, College of Food Science and Engineering, Nanjing University of Finance and Economics, Nanjing 210023, China
[4] Department of Dairy and Food Science and Technology, Egerton University, Egerton 20115, Kenya

S1. Results

S1.1 Effects of different treatment voltages on the whiteness and sterilization rate of A. bisporus.

As shown in Fig. S2 A and C, with the increase of cold plasma treatment voltage, when the treatment voltage was greater than 140 kV, *A. bisporus* showed significant discoloration ($P < 0.05$). When the treatment voltage was 155 kV, the L * of *A. bisporus* was the lowest, which was 74.71. In addition, after cold plasma treatment with different voltages, the total number of colonies and sterilization rate of *A. bisporus* in the treatment group were significantly different from those in the control group (P<0.05).

The sterilization rate of *A. bisporus* was the highest when treated at 155 kV. When the treatment voltage was 80 kV, the impact of cold plasma treatment voltage on the color and sterilization rate of *A. bisporus* was comprehensively evaluated. From this, it could be determined that the best single factor experimental treatment voltage of cold plasma was 80 kV.

S1.2 Effects of different treatment frequencies on the whiteness and sterilization rate of A. bisporus.

As shown in Fig. S3 A and C, when the treatment frequency reached 195 Hz, the most severe browning occurred in *A. bisporus* (P<0.05). This was because high-frequency cold plasma treatment releases a large amount of active substances, causing the phenolic substances on the surface of *A. bisporus* to change, resulting in an enzymatic browning reaction, causing browning on the surface of *A. bisporus*. When the treatment frequency was below 170 Hz, the L^* value of *A. bisporus* did not change

significantly (P>0.05), and no obvious browning occurred in *A. bisporus*.

As shown in Fig.S3 B, after cold plasma treatment with different frequencies, the total number of colonies and sterilization rate of *A. bisporus* in the treatment group were significantly different from those in the control group (P<0.05). When the treatment frequency was 195 Hz, the total number of colonies on the surface of *A.bisporus* reached the lowest, 3.8 lg CFU g^{-1}, and the sterilization rate was 92.12 %.

When the treatment frequency at 120 Hz and after comprehensively evaluating the impact of cold plasma treatment on the color and sterilization rate of *A. bisporus*, and taking into account cost savings and power consumption reduction, the best single factor experimental treatment frequency of cold plasma was determined to be 120 Hz.

S1.3 Effects of different processing times on the whiteness and sterilization rate of A. bisporus.

As shown in Fig. S4 A and C, the results showed that when the processing time reached 13 and 16 minutes, significant browning occurred on the surface of *A. bisporus* (P<0.05). When the processing time was less than 10 min, no obvious browning occurred on the surface of *A. bisporus* (P＞0.05).

This study tested the sterilization effect of cold plasma by measuring the effects of different processing times on microorganisms on the surface of *A. bisporus*. As shown in Fig. S4 B, after being treated with cold plasma for different times, the sterilization rate of the group lasting more than 7 minute was significantly different from that of the control group (P<0.05). After 16 minutes of cold plasma treatment, the total number of bacterial colonies was a minimum of 4.13 lg CFU g^{-1}, and the sterilization rate was

78.70%.

The effects of cold plasma treatment on the color and sterilization rate of *A. bisporus* were comprehensively evaluated. Considering the time cost saving and efficiency improvement, the optimal single factor experimental treatment time of cold plasma was determined to be 7 min.

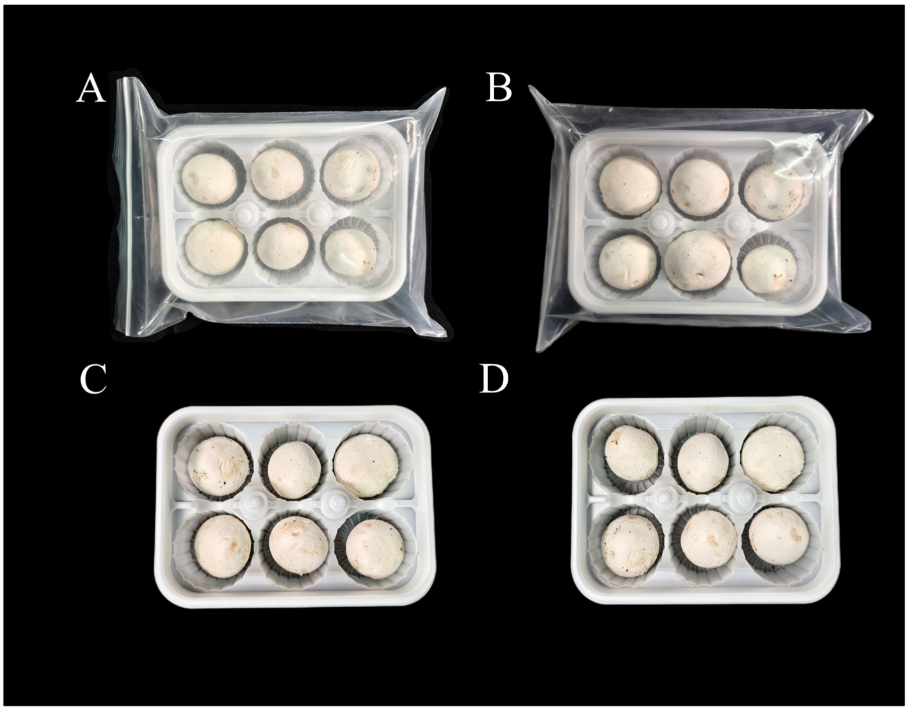

Figure S1. Schematic diagram of *Agaricus bisporus* storage box. (A) Cold plasma treatment combined with PE sealed packaging (CP+PE), (B)PE sealed packaging (PE), (C) direct cold plasma treatment (CP), (D) Control group (Control)

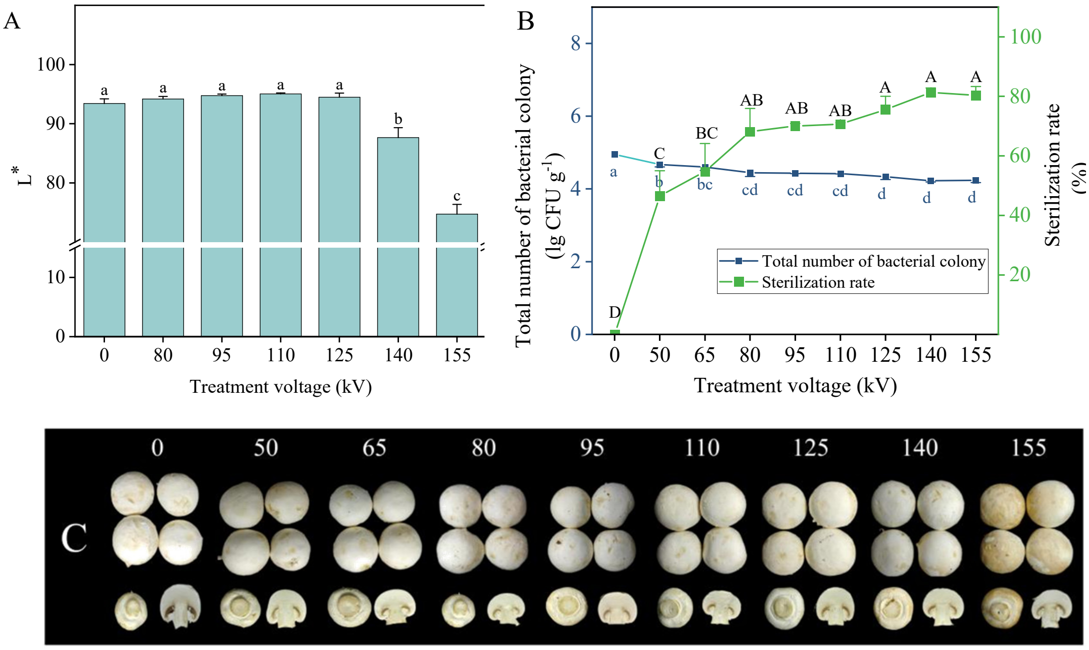

Figure S2. Effects of different treatment voltages on the whiteness (A), total number of colonies and sterilization rate (B), appearance (C) of *Agaricus bisporus*

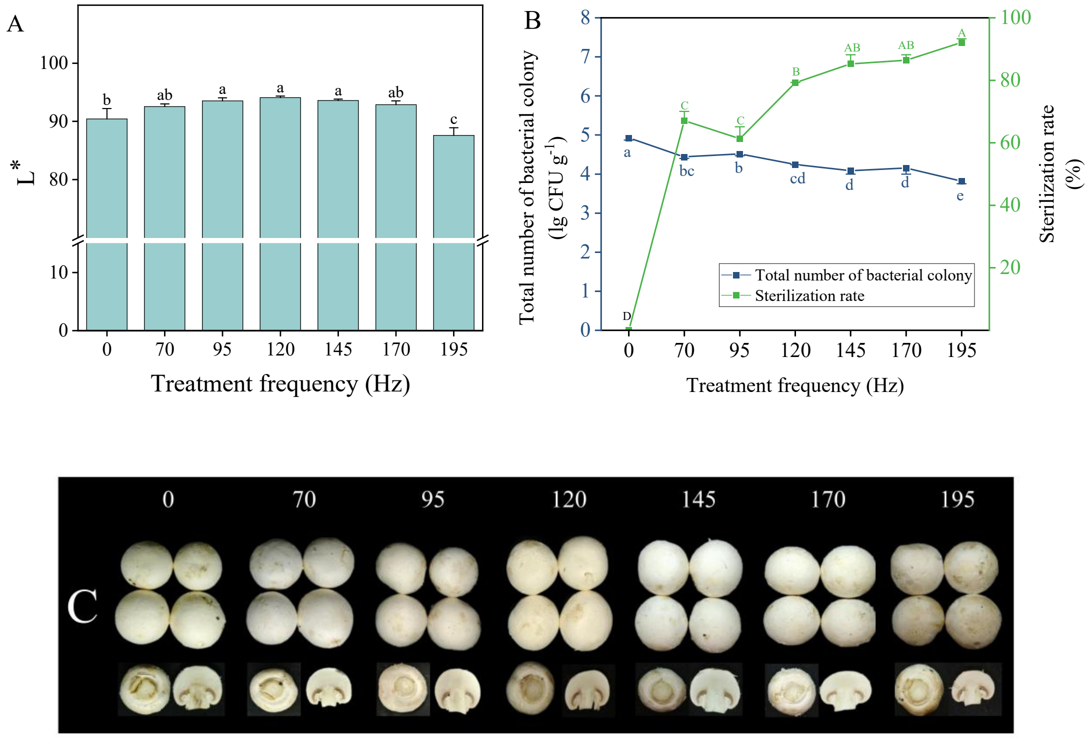

Figure S3. Effects of different treatment frequencies on the whiteness (A), total number of colonies and sterilization rate (B), appearance (C) of *Agaricus bisporus*

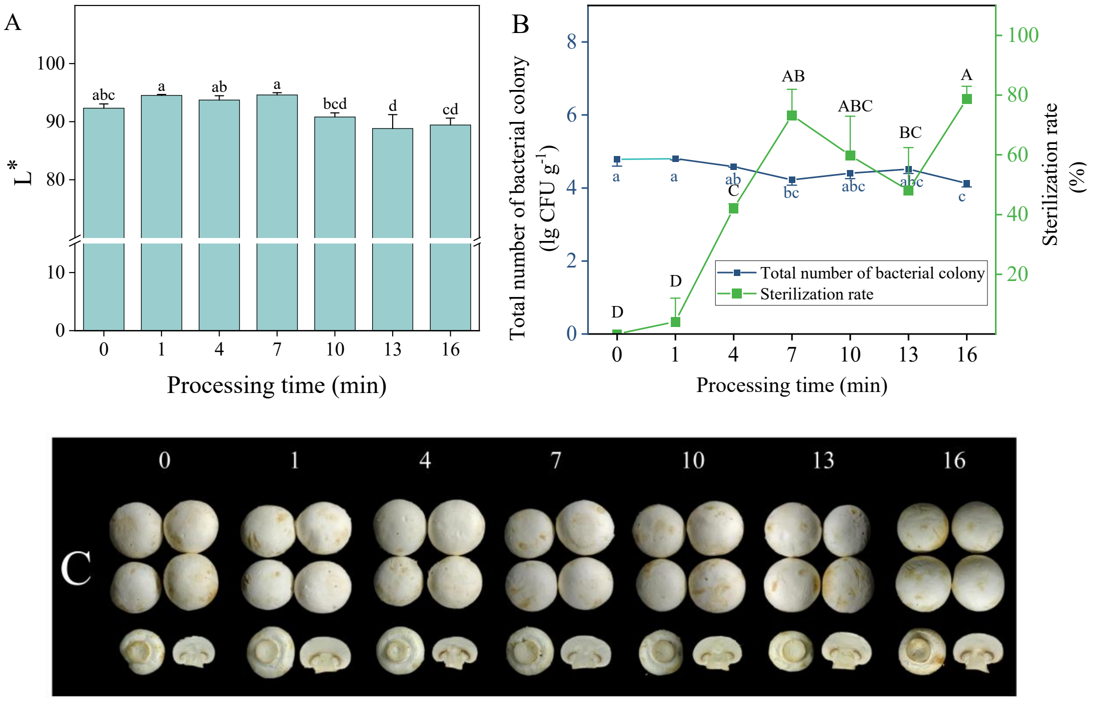

Figure S4. Effects of different processing time on the whiteness (A), total number of colonies and sterilization rate (B), appearance (C) of *Agaricus bisporus*

Table S1. Experiment design and results for response surface analysis

**Experimental group**	**Treatment voltage**	**Treatment frequency**	**Processing time**	**L^***	**Y(%)**
1	80	145	4	92.7546	58.66
2	95	120	10	94.0336	87.09
3	80	120	7	93.6914	74.35
4	80	120	7	93.8125	83.01
5	80	120	7	92.4626	79.41
6	80	95	4	93.4369	54.41
7	80	95	10	92.7617	71.90
8	95	120	4	93.4489	72.55
9	80	145	10	93.5189	90.03
10	65	95	7	93.1569	66.99
11	95	95	7	93.2743	75.65
12	65	120	10	92.0215	80.23
13	95	145	7	94.0464	86.27
14	65	120	4	92.7319	56.54
15	80	120	7	91.2463	79.90
16	65	145	7	92.3482	77.94
17	80	120	7	93.4537	82.68
